# Development and Investigation of a New Polysulfone Dialyzer with Increased Membrane Hydrophilicity

**DOI:** 10.3390/membranes15050132

**Published:** 2025-04-30

**Authors:** Adam M. Zawada, Bettina Griesshaber, Bertram Ottillinger, Ansgar Erlenkötter, Nathan Crook, Skyler Boyington, Manuela Stauss-Grabo, James P. Kennedy, Thomas Lang

**Affiliations:** 1 Product Development, Fresenius Medical Care Deutschland GmbH, 66606 Sankt Wendel, Germany; adam.zawada@freseniusmedicalcare.com; 2 Clinical Research, Global Medical Office, Fresenius Medical Care Deutschland GmbH, 61352 Bad Homburg, Germany; bettina.griesshaber@freseniusmedicalcare.com (B.G.); manuela.stauss-grabo1@freseniusmedicalcare.com (M.S.-G.); 3 Ottillinger Life Sciences, 85649 Brunnthal, Germany; bertram.ottillinger@vicron.com; 4 Biosciences, Fresenius Medical Care Deutschland GmbH, 66606 Sankt Wendel, Germany; ansgar.erlenkoetter@freseniusmedicalcare.com; 5 Applications Laboratory, Fresenius Medical Care, Ogden, UT 84404, USA; nathan.crook1@freseniusmedicalcare.com (N.C.); skyler.boyington@freseniusmedicalcare.com (S.B.); 6 Product Development, Fresenius Medical Care, Ogden, UT 84404, USA; james.kennedy@freseniusmedicalcare.com

**Keywords:** dialyzer, membrane, hemocompatibility, performance, hydrophilicity, end stage kidney disease

## Abstract

Innovation in dialysis care is fundamental to improve well-being and outcomes of patients with end-stage kidney disease. The dialyzer is the core element of dialysis treatments, as it largely defines which substances are removed from the patient’s body. Moreover, its large surface size is the major place of interaction of the patient’s blood with artificial surfaces and thus may lead to undesired effects such as inflammation or coagulation. In the present article we summarize the development path for a new dialyzer, including in vitro and clinical evidence generation. We use the example of the novel FX CorAL dialyzer, which has recently entered European and US markets, to show which steps are needed to develop and characterize a new dialyzer. The FX CorAL dialyzer includes a new hydrophilic membrane, which features reduced protein adsorption, sustained performance, and an improved hemocompatibility profile, characterized in numerous in vitro and clinical studies. Safety evaluations revealed a favorable profile, with low incidences of adverse device effects. Insights gained from both in vitro and clinical studies contribute to the advancement of dialyzer development, ultimately leading to improved patient care.

## 1. Introduction

Hemodialysis is a life-saving treatment for millions of patients with end-stage kidney disease (ESKD) [1,2]. The core element of such treatments is the dialyzer, which replaces the function of the kidney during a ~4 h session, generally three times a week. In contrast to a continuously working kidney of healthy persons, the dialyzer has to eliminate accumulated uremic toxins in a very limited time frame from the patient’s body. Thus, the performance of a dialyzer must approach the selective cleaning characteristics of a healthy human kidney, i.e., removal of small and middle molecules, while preserving essential proteins such as albumin. Moreover, during dialysis treatment, patient’s blood contacts artificial surfaces, such as the capillary fibers, which potentially leads to an activation of different blood components, such as immune cells, platelets, or the complement system, contributing to undesirable outcomes such as inflammation or coagulation/clotting.

Thus, during the development of a new dialyzer, different aspects of performance and hemocompatibility must be considered to achieve optimal blood-purification properties and the reduction of short- and long-term adverse events in ESKD patients. Therefore, thoughtful design, together with excessive in vitro and clinical testing, are necessary to achieve a high-performing and safe product.

Taking the example of the novel FX CorAL dialyzer (Fresenius Medical Care, Bad Homburg, Germany), this article summarizes the development path of a new polysulfone dialyzer, including the scientific evidence-generation stream alongside the development to characterize performance, hemocompatibility, and safety of this new dialyzer.

## 2. Development of a New Dialyzer

A dialyzer consists of many parts, including the housing, flange, tubing connectors, potting, and the core element—the membrane, which mainly defines the dialyzer’s performance and hemocompatibility features. While the housing, flange, and tubing connectors mainly represent the outer dialyzer design, the membrane and potting are located within the dialyzer.

### 2.1. Outer Dialyzer Design

**Figure 1:**
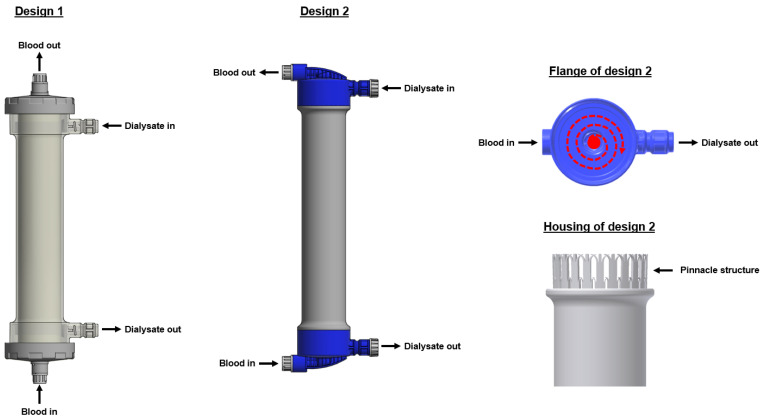
Outer dialyzer designs. Design 1 with blood inlet and outlet in vertical direction; Design 2 with inlet and outlet in horizontal direction, and dialysate ports within the flanges. The flange of design 2 leads to a spiral blood flow while the pinnacle structure within the housing allows dialysate entering from all sides and prevents detachments of the potting from the housing.

With regards to the outer dialyzer design, two main designs are currently available (Figure 1). In the first variant, the blood enters and leaves the flanges in a vertical direction. Moreover, the dialysate tubing connections are located within the housing of the dialyzer. In the second design, both the blood and dialysate tubing connectors are located within the flanges, and the connections are in a horizontal direction. This design with lateral blood-inlet ports leads to a spiral and homogenous blood-flow path within the flange and may increase the performance of the dialyzer [3,4,5,6]. Moreover, in contrast to the first design, the dialysate distribution may be more homogeneous as the dialysate does not only enter the fiber compartment from one side, but the additional pinnacle structure of the housing supports the dialysate to enter the fiber compartment from all sides at a similar flow rate [4,5,6]. Moreover, the pinnacle structure prevents the detachment of the potting from the housing, which may happen with dialyzers of design 1, especially when exposed to higher temperatures. The FX CorAL dialyzer uses the second dialyzer design.

Moreover, different materials can be utilized for the dialyzer outer parts. The two main materials are polycarbonate and polypropylene. One advantage of polypropylene is the lower weight, which has positive environmental aspects during, e.g., transportation and disposal. While dialyzers of design 1 are available with polycarbonate or polypropylene housings and flanges, the second dialyzer design exclusively uses polypropylene housings and flanges.

### 2.2. Dialyzer Membrane

Within the dialyzer, the fiber bundle, containing ~5.000–20.000 single capillary fibers, is potted at the two ends to allow separation of the blood and dialysate compartment. Based on the number of fibers, as well as the fiber length and geometry, the effective surface size can be calculated for each dialyzer. For the FX CorAL, nine different models are available, with surface sizes ranging from 0.6 m^2^ to 2.5 m^2^. Moreover, different fiber geometries are available for the FX CorAL with an inner diameter of 185 µm and wall thickness of 35 µm for the models FX CorAL 40 (0.6 m^2^), FX CorAL 50 (1.0 m^2^), FX CorAL 60 (1.4 m^2^), FX CorAL 80 (1.8 m^2^), FX CorAL 100 (2.2 m^2^) and FX CorAL 120 (2.5 m^2^), as well as an inner diameter of 210 µm and wall thickness of 40 µm for the models FX CorAL 600 (1.6 m^2^), FX CorAL 800 (2.0 m^2^) and FX CorAL 1000 (2.3 m^2^), the latter three models optimized for hemodiafiltration (HDF) treatments. Due to the different surface sizes and geometry, the models also differ in their performance characteristics such as clearance values or ultrafiltration coefficients (e.g., ultrafiltration coefficient FX CorAL 40: 28 mL/h/mmHg, FX CorAL 120: 94 mL/h/mmHg, FX CorAL 600: 60 mL/h/mmHg, FX CorAL 1000: 82 mL/h/mmHg).

The hollow-fiber membranes may consist of different materials based on cellulose or synthetic polymers such as polysulfone, poly(aryl)ethersulfone, polyphenylene, acrylonitrile, polyester-polymer alloys, or polymethylmethacrylate [6,7,8,9]. Synthetic dialyzer membranes are most widely used in clinical practice. It is important to note that, although different dialyzers with the same base-material polysulfone exist on the market, continuous development of the polysulfone membrane during the last decades led to significant differences in the currently available polysulfone-based dialyzers. This development includes many different aspects during manufacturing and sterilization, leading to differences in, e.g., fiber geometry (e.g., inner diameter, wall thickness), pore size distribution, or hydrophilicity. One of the most important, and yet intransparent, attributes of polysulfone membranes is the concentration and distribution of polyvinylpyrrolidone (PVP). PVP is added to the membrane spinning mass as a hydrophilic agent, given that polysulfone is hydrophobic and increased membrane hydrophilicity is associated with reduced membrane fouling, improved hemocompatibility, and sustained performance [10,11,12,13,14,15,16,17,18,19,20,21].

For the production of standard hemodialysis membranes, a spinning solution, consisting of a polymer (e.g., polysulfone), co-polymer (PVP), and solvent, is run through the outer part of the spinneret, while the precipitation fluid (non-solvent) passes the spinneret through the inner orifice leading to the formation of the hollow fiber, which is then solidified in a bath containing the non-solvent [22]. During the development of the FX CorAL membrane (Helixone *hydro*), the spinning process was refined in a way, in that PVP was additionally added to the precipitation fluid leading to a PVP-enriched blood-side surface. Moreover, small amounts of the antioxidant α-tocopherol were added to the spinning solution, to prevent oxidation and elution of PVP during shelf life [10,13]. The increased hydrophilicity of the blood-side surface is important given that the inner membrane surface is the major place of interaction of the patient’s blood during the extracorporeal treatment. The repulsive hydration force of the hydrophilic membrane reduces the interaction and adsorption of blood components, such as proteins, with the membrane and thus improves hemocompatibility [10,13,14]. Proteins generally bind to such artificial surfaces and undergo structural changes that subsequently trigger immune system activation [23]. Besides the FX CorAL dialyzer, another dialyzer with hydrophilic membrane modification is available on the market. The Toraylight NV (Toray Medical, Tokyo, Japan) with the “Hydrolink membrane” uses a hydrophilic polymer to make its polysulfone membrane hydrophilic [6,24,25,26,27,28]. In line, also this hydrophilic modification has been associated with positive hemocompatibility effects, including antithrombotic activity [24,26,28].

PVP stability is another aspect that differs among different dialyzers used in clinical practice. PVP may undergo oxidation, chain breaks, and subsequent elution from the membrane, which then may lead to a reduction of the positive effects of this compound. Generation of elutable PVP may occur either during manufacturing by, e.g., using radiation as a sterilization process or during shelf life as a continuous slow oxidation process. During the FX CorAL development and production, both PVP oxidation risks were reduced: (1) The FX CorAL sterilization process uses INLINE steam sterilization where no radiation is applied, but hot steam and sterile water are continuously rinsed through the dialyzer. (2) To reduce oxidation of PVP over time, small amounts of the anti-oxidant α-tocopherol are added to the spinning mass, leading to a stabilization of PVP over the complete shelf life [10].

## 3. Investigation of a New Dialyzer

### 3.1. Membrane Characterization

#### 3.1.1. Characterization of PVP Content and Elution

To investigate whether the addition of PVP into the precipitation fluid during spinning leads to an increased amount of PVP on the blood-side surface, the Helixone *hydro* membrane was tested with X-ray photoelectron spectroscopy (XPS), which allows quantification of the atomic composition just of the fiber surface [13]. When compared to the predecessor membrane Helixone *plus* of the FX CorDiax (Fresenius Medical Care, Bad Homburg, Germany), which does not have additional PVP in the precipitation fluid during spinning, the novel Helixone *hydro* membrane had significantly higher PVP content on the blood-side surface (FX CorAL: 26.3% mass, FX CorDiax: 23.0%, *p* < 0.05). This was also the case when comparing to other polysulfone- or polyethersulfone-based membranes (xevonta [B.Braun, Melsungen, Germany]: 24.7%, *p* = 0.356; ELISIO [Nipro, Osaka, Japan]: 24.4%, *p* = 0.219; Polyflux [Baxter, Deerfield, IL, USA]: 18.3%, *p* < 0.001; Theranova [Baxter, Deerfield, IL, USA]: 15.6%, *p* < 0.001). Moreover, in recirculation experiments up to 10 h, no elutable PVP from the Helixone *hydro* membrane was detected (below quantification limit of 0.5 mg/1.6 m^2^ dialyzer surface). In contrast, other polysulfone- and polyethersulfone-based membranes, sterilized with gamma (xevonta: 3.2 mg/1.6 m^2^, *p* < 0.001; ELISIO: 2.1 mg/1.6 m^2^, *p* < 0.001) or autoclave steam (Polyflux: 9.0 mg/1.6 m^2^, *p* < 0.001; Theranova: 9.1 mg/1.6 m^2^, *p* < 0.001), showed a strong elution of PVP already after 5 h [13]. In addition, the generation of elutable PVP was investigated also over the complete shelf-life of 3 years [10]. Also in these experiments, no eluted PVP was detected from the Helixone *hydro* membrane after 3 years, supporting the stabilizing effect of α-tocopherol [29].

These results were in line with the measurements of the surface charge of the dialyzer membranes [14]. Zeta potential was determined for the FX CorAL and other polysulfone-, polyethersulfone- and cellulose-based dialyzers. Here, the FX CorAL showed the most neutral surface charge (−2.38 mV) as compared to all other investigated dialyzers (FX CorDiax: −5.56 mV, *p* < 0.001; xevonta: −7.30 mV, *p* < 0.001; Polyflux: −7.15 mV, *p* < 0.001; ELISIO: −7.12 mV, *p* < 0.001; FDX [Nikkiso, Tokyo, Japan]: −25.74 mV, *p* < 0.001; Theranova: −9.39 mV, *p* < 0.001; Sureflux [Nipro, Osaka, Japan]: −6.28 mV, *p* < 0.001).

#### 3.1.2. Characterization of Membrane Hydrophilicity

It is well-established that increased PVP content on the membrane surface leads to increased hydrophilicity [15,20]. This association was also confirmed for the FX CorAL with contact angle measurements, where fibers are placed in a dye-containing water reservoir and the capillary height is measured after a predefined time [10]. The contact angle is then determined based on the capillary height and additional information such as the membrane geometry. When two membranes with the same geometry but different hydrophilicity are compared, the more hydrophilic membrane will have a lower contact angle than the less hydrophilic membrane. In these experiments, the Helixone *hydro* membrane of the FX CorAL dialyzer had a significantly lower contact angle then the predecessor membrane Helixone *plus* of the FX CorDiax dialyzer (FX CorAL: 45.1°, FX CorDiax: 51.7°, *p* < 0.001), confirming higher hydrophilicity of this novel membrane [10].

**Figure 2:**
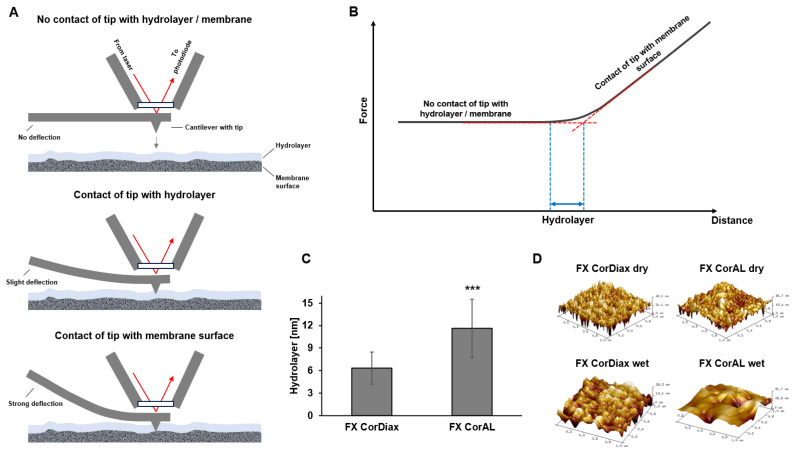
Measurement of hydrolayer. (**A**) Atomic force microscopy (AFM) determination of hydrolayer thickness. Membranes are cut open and exposed to water. Contact of the tip to the hydrolayer and membrane surface leads to a deflection of the cantilever. (**B**) For analysis, deflection of the cantilever is plotted in a force-distance diagram and the curved portion represents the thickness of the hydrolayer. (**C**) FX CorAL membrane shows a significantly thicker hydrolayer than the FX CorDiax (*n* = 30 each, *** *p* < 0.001). (**D**) AFM characterization of membrane surface in dry and wet conditions for FX CorDiax and FX CorAL.

These results were confirmed by atomic force microscopy (AFM) measurements of the hydrolayer thickness. For the measurement, a fiber is cut lengthwise and affixed on a slide with the inner lumen exposed. A water droplet is placed on the fiber and on the AFM probe mount window. The two droplets are then brought together to form a continuous water bridge. AFM uses a cantilever with a tip that taps or moves on the membrane surface (Figure 2A). The cantilever bends to a different degree when the tip comes into contact with the hydrolayer or the membrane surface, leading to a reflection of the laser at different angles. These laser signals are measured and translated into a force-distance curve (Figure 2B). The force-distance curve comprises three different sections: (1) the approach section without increasing force, corresponding to the first step where the tip has no contact with the hydrolayer or the membrane and thus the cantilever shows no deflection, (2) a non-linear section, corresponding to the contact of the tip with the PVP-water gel (“hydrolayer”) and (3) a final linear section with increasing force, corresponding to the contact of the tip with the solid membrane surface and the strong deflection of the cantilever. Based on this curve, the thickness of the hydrolayer can be determined as displayed in Figure 2B. These results showed that the Helixone *hydro* membrane of the FX CorAL has a significantly thicker hydrolayer as compared to the Helixone *plus* membrane of the FX CorDiax (Figure 2C), in line with the increased PVP content on the blood-side surface and the lower contact angle, showing that the AFM determination of hydrolayer thickness is a good measure of membrane hydrophilicity [10,13]. AFM also allows generation of 3D surface images of dry and wet membranes, showing that the FX CorAL membrane becomes smoother than the FX CorDiax under wet conditions (FX CorAL: Ra = 5.3; FX CorDiax Ra = 11.8) (Figure 2D). Lower membrane roughness is associated with positive effects, such as lower protein adsorption and blood cell interaction [30,31].

#### 3.1.3. Characterization of Membrane Fouling

Many previous studies demonstrated that increased membrane hydrophilicity, achieved via increased PVP content, reduces protein adsorption to the membrane via repulsive hydration force [15,16,17,18,19,20]. Different experimental approaches were applied to test whether also the Helixone *hydro* membrane of the FX CorAL dialyzer also shows lower secondary membrane formation than other membranes. In the first experiment, the sieving coefficient of albumin was investigated over time [14]. As proteins from the recirculated plasma adsorb to the membrane, the sieving coefficient decreases over time, especially in the first 15 min of plasma contact with the membrane. This is due to a reduction of the effective pore size by the generated protein layer [10]. These experiments found that the FX CorAL had the lowest reduction of the albumin sieving coefficient over time, pointing towards lowest secondary membrane formation. In the second experiment, the increase of the transmembrane pressure (TMP) at constant inlet and filtrate flows in a plasma recirculation experiment was investigated over time. This is also a good marker of secondary membrane formation, given that with an additional protein layer, more pressure is needed to achieve the same filtrate flow through the membrane. Also in this experiment, the FX CorAL showed the lowest increase of the TMP over time, especially in the first minutes of recirculation [12]. These results were also confirmed with other protein-containing solutions, showing generally low protein binding by the Helixone *hydro* membrane [10,11].

### 3.2. In Vitro and Clinical Investigation of Performance, Hemocompatibility and Safety

#### 3.2.1. Overview of In Vitro and Clinical Studies with the New Polysulfone Dialyzer

Beyond the necessary performance and hemocompatibility testing, as requested by respective standards, such as ISO 8637 or ISO 10993, and the information which has to be presented in the instructions for use (IFU), more detailed investigations may allow a better understanding of the new product. For example, the clearance values presented in the IFU are measured in an aqueous solution. However, in clinical use the performance changes over treatment time due to protein adsorption to the dialyzer membrane [10,12,32,33,34,35,36,37,38]. Therefore, in vitro and clinical investigation of performance should take such considerations into account.

Such in vitro and clinical studies were also performed for the FX CorAL dialyzer. This chapter summarizes and analyzes these in vitro [10,11,12,13,14] and clinical studies [39,40,41,42] regarding the key dialyzer features. Moreover, Table 1 and Table 2 give an overview of these in vitro and clinical studies, respectively.

#### 3.2.2. Investigation of Performance

##### Importance of a High-Performing Dialyzer

The major function of the dialyzer is the removal of uremic toxins and excess water from patients with ESKD. These uremic toxins have a broad molecular weight range and have been associated with different comorbidities such as inflammation, malnutrition or cardiovascular disease [43,44,45,46,47]. This is especially the case for so-called middle molecules such as β2-microglobulin (~12 kDa), as higher levels have been linked to reduced survival among dialysis patients [46,48,49]. Therefore, efficient removal of such toxins during dialysis is fundamental for the well-being of patients with ESKD. Moreover, besides the efficient removal of a broad range of uremic toxins, the dialyzer shall have a sharp molecular weight cut-off to prevent significant loss of essential proteins, such as albumin. Reduced serum albumin concentration contributes to protein-energy wasting in dialysis patients and is a strong predictor of mortality [50,51,52].

Furthermore, while manufacturers present dialyzer performance values, such as clearances, in the respective IFUs, it is important to understand that dialyzer performance is not constant but may change during a dialysis session. This is mainly due to the formation of a secondary membrane as adsorption of plasma proteins to the dialyzer membrane reduce the effective pore size [11,12,34]. This effect of reduced performance is predominant for larger proteins than for smaller solutes such as urea, creatinine or vitamin B12 [11,12,34,35,36,37].

Therefore, in vitro and clinical testing during the development and investigation of a new dialyzer shall take all these considerations into account. The following two chapters summarize the in vitro and clinical studies regarding performance characterization of the FX CorAL dialyzer.

##### Investigation of Performance in In Vitro Studies

Beyond the standard performance testing for the IFU, including measurements of clearances at different flow rates (e.g., urea, creatinine, phosphate, vitamin B12, cytochrome C), sieving coefficients (e.g., β2-microglobulin, myoglobin, albumin), dialyzer mass transfer-area coefficient (K0A), ultrafiltration coefficients or pressure drop at the blood compartment, further in vitro testing was performed to characterize the impact of hydrophilic membrane modification of the FX CorAL with regard to performance stability over treatment time.

First, in contrast to the IFU sieving coefficient data, which are determined typically after 30 min of plasma recirculation, time-dependent characterization of the β2-microglobulin, myoglobin, and albumin sieving coefficients up to 240 min was performed [12,14]. In these investigations, sieving coefficients for all three proteins decreased over time, with the strongest decrease in the first ~20 min of plasma recirculation and with a higher magnitude for larger (i.e., albumin, 68 kDa) than for smaller (i.e., β2-microglobulin, 12 kDa) proteins. In these experiments, the FX CorAL had the lowest reduction of the sieving coefficients as compared to other synthetic dialyzers, pointing towards lower secondary membrane formation due to increased membrane hydrophilicity. Based on these time-dependent sieving coefficient data, molecular weight retention curves were generated, which showed the lowest shift over time towards lower Stokes radius for the FX CorAL, as compared to the other investigated dialyzers (FX CorAL: −0.23 nm at sieving coefficient of 50%; ELISIO: −0.29 nm; xevonta: −0.50 nm) [12]. These curves also allowed to determine the molecular weight cutoff (MWCO, molecular weight or Stokes radius at sieving coefficient of 10%) and molecular weight retention onset (MWRO, molecular weight or Stokes radius at sieving coefficient of 90%) over time. For all investigated time points, the FX CorAL showed the steepest molecular weight retention curve, leading to the lowest difference between MWCO (2 min: 2.66 nm; 120 min: 2.51 nm) and MWRO (2 min: 1.96 nm; 120 min: 1.65 nm), as compared to the other investigated dialyzers in this study. This supports the selective permeability of the FX CorAL membrane, with high permeability for middle molecules while preventing significant loss of essential proteins, such as albumin, in parallel. In line with these data, the mean effective pore radius declined to a lower extent for the Helixone *hydro* membrane of the FX CorAL, as compared to the other investigated dialyzers [12]. When, for example, comparing the pore distribution of the FX CorAL and xevonta dialyzers before and after protein fouling in relation to the Stokes radius of β2-microglobulin, the percentage of pores with an effective radius greater than the Stokes radius of β2-microglobulin before protein fouling was much higher (2 min: FX CorAL: 98.7%; xevonta: 97.2%) than after protein fouling (120 min: FX CorAL: 92.4%; xevonta: 85.8%), with a lower decrease for the FX CorAL dialyzer [11]. Interestingly, the performance for the small molecules urea and creatinine remained stable throughout the investigated time (60 min) for all dialyzers, supporting previous studies, that secondary membrane formation especially impacts the elimination of larger molecules [11,34,35,36,37].

Second, in recirculation experiments with human plasma and other protein-containing solutions, the impact of secondary membrane formation on membrane filtration characteristics was investigated [11,12]. Typically, due to the additional protein layer, which is formed during treatment, more pressure is needed to achieve the same filtration flow through the dialyzer membrane [7,11,12]. In recirculation experiments with constant inlet and filtration flows, the FX CorAL showed the lowest TMP increase throughout the experiment, as compared to all other investigated dialyzers, pointing towards lowest secondary membrane formation [11,12]. This was especially the case in the first ~30 min, where the protein fouling process is most active. Vice versa, when investigating the reduction of filtration flow at constant TMP, the FX CorAL showed the most stable filtration rate, which may help to achieve high exchange volumes during hemodiafiltration treatments [11], recently shown to improve mortality among dialysis patients as compared to standard high-flux hemodialysis treatments [53].

Third, in an in vitro study the potential albumin loss by the FX CorAL over a 240-min treatment was compared to other dialyzers [12]. In line with the lowest initial albumin sieving coefficient of the FX CorAL, this dialyzer showed a low level of albumin loss throughout the total duration [12,14].

##### Investigation of Performance in Clinical Studies

Four clinical studies investigated the performance of FX CorAL in a randomized controlled setting (Table 2) [39,40,41,42]. Table 3 presents an overview of patient characteristics and treatment parameters. The main difference in the treatment parameters between the four clinical studies was the use of autosub plus in the comPERFORM study, leading to higher substitution and, accordingly, higher convection volumes. When comparing the treatment parameters to the recommendation of ≥23 L convection volume during a HDF treatment, which has recently been associated with improved patient survival [53], comPERFORM and eMPORA III reached this threshold.

All four studies investigated β2-microglobulin removal as the primary endpoint. Moreover, removal rates and clearances of other uremic toxins of different molecular ranges were also investigated in all studies. Albumin loss into the dialysate was investigated in the comPERFORM study only (Table 2). The results for the comparisons between dialyzers are summarized in Table 4.

**Figure 3:**
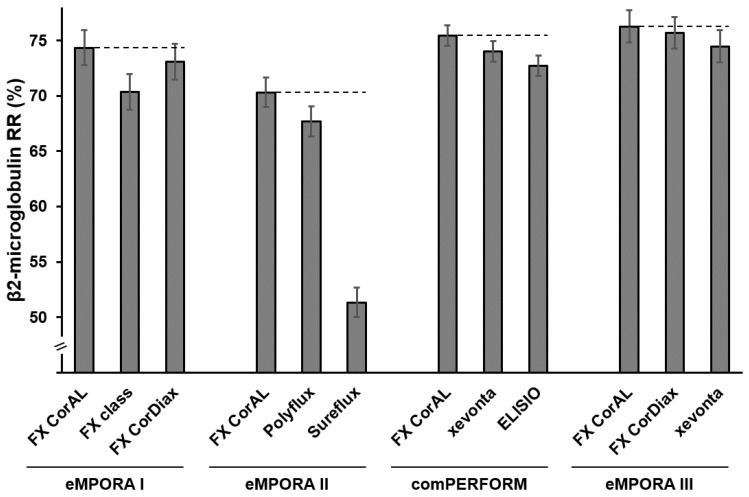
β2-microglobulin removal rates (least square mean ± standard error) of FX CorAL and comparators in the four randomized controlled trials eMPORA I, eMPORA II, comPERFORM, and eMPORA III [39,40,41,42].

Across all four clinical studies, the FX CorAL showed the highest β2-microglobulin removal rate as compared to all other investigated dialyzers (Figure 3). Statistical analysis of non-inferiority and superiority testing is given in Table 4, showing that the predefined endpoints were reached. While these differences appear numerically low, it has to be taken into account that these results were obtained from a single treatment and these small differences during the repeating dialysis sessions may have a meaningful clinical impact on the patients over time. Longer clinical studies are needed to investigate this performance improvement relative to patient morbidity and mortality rates.

When comparing the β2-microglobulin removal rates across the four clinical studies, eMPORA I and II achieved lower removal rates, as compared to eMPORA III and comPERFORM, which applied higher convection volumes.

Moreover, it is important to mention that these clinical results for the β2-microglobulin removal rate are in line with the in vitro investigations of the FX CorAL dialyzer [12]. Also here, the FX CorAL showed the highest β2-microglobulin sieving coefficient values throughout the experiment (2 min: 98.8%, 120 min: 91.6%) when compared to the ELISIO (2 min: 91.0%, 120 min: 84.0%) and xevonta (2 min: 96.3%, 120 min: 87.6%) dialyzers. To obtain high removal rates during treatment, it is crucial that the dialyzer membrane sustains the selective permeability throughout the treatment, which is achieved by the FX CorAL due to lower protein absorption to its membrane.

The results for the secondary endpoints in the four clinical studies regarding removal rates and clearances of middle molecules (β2-microglobulin, myoglobin) were in line with the primary endpoint, reflecting the optimization of the new Helixone *hydro* membrane of FX CorAL dialyzer for middle molecule removal. Regarding removal rates and clearances of smaller molecules, such as phosphate, creatinine, and urea, the results were mostly comparable between the different investigated dialyzers.

The comPERFORM study also investigated the albumin loss during the dialysis session by measuring the albumin concentration in the spent dialysate. Here, the FX CorAL demonstrated the lowest amount of albumin loss up to the 60-min time point. Albumin sieving of FX CorAL remained almost constant thereafter, whereas the other dialyzers’ sieving rates showed a change of the sieving kinetics. These data are in line with in vitro examinations indicating lower secondary membrane formation by protein adsorption on the Helixone *hydro* membrane [14].

Besides the eMPORA and comPERFORM studies, two further clinical studies by other groups also investigated the performance of the FX CorAL dialyzer in high-volume post-dilution HDF treatments [27,54]. The first study was a prospective study with 19 patients, comparing different models of FX CorAL and FX CorDiax (surface size between 1.4 m^2^ and 2.0 m^2^) [54]. Beyond the performance markers investigated in the eMPORA and comPERFORM studies, this study additionally investigated the reduction ratios (RR) of the larger kappa free immunoglobulin light chains (22.5 kDa), prolactin (23 kDa), α1-microglobulin (33 kDa), α1-acid glycoprotein (41 kDa), lambda-free immunoglobulin light chains (45 kDa), as well as albumin RR and loss (68 kDa). No significant differences were found for the small, middle, and larger molecules when comparing the FX CorAL and FX CorDiax with the same surface size, except for the lambda-free immunoglobulin light chains, where FX CorDiax 80 had significantly higher RR than FX CorAL 80. Also, no differences were found in terms of albumin loss. These results are in line with the eMPORA I and eMPORA III studies, which also compared the FX CorAL and FX CorDiax dialyzers (FX CorAL 600 vs. FX CorDiax 600) and found slightly higher removal rates for the FX CorAL dialyzer (Figure 3) (ß2-microglobulin RR from Maduell et al. [54]: FX CorAL 600: 85.4%, FX CorDiax 600: 84.1%). As these differences are not significantly different, it has to be noted that the development of the FX CorAL aimed to improve hemocompatibility by reducing protein adsorption to the membrane. Indeed, reduced fouling is also associated with more stable performance during treatment, but also other parameters such as pore size distribution determine the overall dialyzer performance.

The second study was also a prospective study with 20 patients, comparing the performance of the Toraylight NV 2.1 dialyzer (Toray Medical, 2.1 m^2^) with the performance of the FX CorAL 800 (2.0 m^2^) dialyzer [27]. The study investigated the same markers as mentioned in the previous study and additionally measured the protein-bound uremic toxins p-cresyl sulfate (188 Da) and indoxyl sulfate (213 Da). No differences in the RR were found for the small molecules, as well as for β2-microglobulin, indoxyl-sulfate, and p-cresyl sulfate. For the larger molecules myoglobin, kappa free immunoglobulin light chains, prolactin, α1-microglobulin, α1-acid glycoprotein, and lambda-free immunoglobulin light chains, the Toraylight NV 2.1 dialyzer showed higher RR than the FX CorAL 800 dialyzer under the same convection conditions. However, under these conditions, a substantial albumin loss over 5 g per session in more than 50% of the patients was observed in treatments with the Toraylight NV 2.1 dialyzer, which was not the case in treatments with the FX CorAL dialyzer (FX CorAL 800: 1.3 g, Toraylight NV 2.1: 6.2 g).

#### 3.2.3. Investigation of Hemocompatibility

##### Importance of a Hemocompatible Dialyzer

Hemocompatibility is an indispensable feature of dialyzer membranes and comprises many complex reactions between blood components and artificial surfaces, especially with the membrane, given its large surface size. These interactions trigger a cascade of immune, coagulative, and inflammatory responses, including complement activation, platelet activation, coagulation, as well as cell activation, inflammation, and oxidative stress [23,55,56].

1. Complement activation

Complement activation is one of the primary immune responses to foreign surfaces. After contact of blood to the dialyzer membrane, complement proteins, especially C3, bind to the surface and undergo activation. This initiates the alternative complement pathway, which is typically the primary pathway in this context [55,56]. However, the lectin pathway and classical pathway can also be involved depending on the membrane’s surface properties [55,56,57,58]. Upon activation, C3 initiates a complement cascade activation, leading to the generation of different complement factors and finally the formation of the cytolytic membrane attack complex (C5b-9, MAC) [23,55,56,59]. Although full C5b-9 formation on cell surfaces in dialysis is rare, sublytic MAC fragments cause cellular activation and inflammation [23,55,56]. During the complement cascade activation and MAC formation, two fragments, C3a and C5a, also known as anaphylatoxins, are released [23,55,56,60]. These small peptides are potent pro-inflammatory molecules that attract and activate leukocytes, contributing to systemic inflammation. Chronic complement activation leads to a constant low-grade inflammatory state in dialysis patients, which contributes to cardiovascular complications, immune suppression, and poor clinical outcomes [23,55,61].

2. Platelet activation and coagulation

Platelet activation and coagulation is a further critical hemocompatibility challenge during the development of new dialyzers, given the thrombogenic potential of dialysis membranes [23,56]. Upon contact to non-endothelial surfaces like dialysis membranes, platelets adhere and become activated [23,56]. This activation is mediated by membrane receptors such as glycoprotein IIb/IIIa (GPIIb/IIIa), which undergo conformational changes that lead to platelet aggregation [21,23,56]. Additionally, complement fragments, especially C3a and C5a, interact with platelets and increase their activation. C5a can bind to platelet receptors, enhancing the release of pro-coagulant molecules and aggregation. Activated platelets release ADP, thromboxane A2, and other pro-thrombotic molecules, creating a positive feedback loop that encourages further platelet activation and thrombus formation on the dialysis membrane [23,56,62]. Beyond platelet activation, hemodialysis induces activation of the plasmatic coagulation system. This is evidenced by increasing levels of thrombin-antithrombin (TAT) complexes, indicating elevated thrombin generation during dialysis treatment [63,64,65]. Increased activation of platelets and the plasmatic coagulation system raise the risk of clot formation in the dialysis circuit, necessitating anticoagulation therapy. However, this also elevates the bleeding risk for patients, creating a complex clinical management challenge.

3. Cell activation, inflammation and oxidative stress

Cell activation during dialysis affects different cell types beyond platelets such as leukocytes or endothelial cells [23,55,56,66,67]. Activated leukocytes release pro-inflammatory cytokines such as TNF-α, IL-1β, and IL-6, as well as reactive oxygen species (ROS), contributing to systemic inflammation [23,55,56]. Increased ROS leads to oxidative stress, which exacerbates inflammation and may damage red and white blood cells, increasing cell fragility and disrupting normal cellular functions [23,55]. Besides ROS, also the anaphylatoxins C3a and C5a may activate different cells in the patient, such as endothelial cells [23,55]. Endothelial activation results in the upregulation of adhesion molecules like ICAM-1 and VCAM-1, facilitating leukocyte adherence and transmigration [23,68]. This response can impair vascular function and contribute to atherosclerosis in chronic dialysis patients [23]. Chronic cell activation in dialysis patients leads to a persistent inflammatory state and oxidative stress, which are linked to complications such as atherosclerosis, cardiovascular disease, anemia, and reduced immune function [23,61].

##### Investigation of Hemocompatibility in In Vitro Studies

During the development of the FX CorAL dialyzer, in vitro hemocompatibility studies were performed to characterize the activation of complement factors C3a, C5a, and sC5b-9 in a recirculation setup with human blood [14]. Here, the FX CorAL showed the lowest complement activation for all factors as compared to seven polysulfone-, polyethersulfone- and cellulose-based dialyzers. While most of the investigated dialyzers showed higher complement activation than the reference dialyzer (FX class, Fresenius Medical Care), which was run in parallel for standardization, the FX CorAL showed a −39.4% [C3a], −57.5% [C5a], and −58.9% [sC5b-9] lower complement activation than the reference dialyzer [14].

In the same recirculation setup with human blood, also platelet loss, as a marker of platelet activation, was investigated [13]. Also here, the FX CorAL showed lower platelet loss than all other investigated dialyzers. When comparing platelet loss to the reference dialyzer, the FX CorAL showed a −225.2% lower platelet loss. This indicates lower activation of platelets by the hydrophilic membrane of the FX CorAL dialyzer.

The results of these in vitro hemocompatibility investigations strongly correlated with the slope of the albumin sieving coefficient decrease over time, supporting previous evidence that protein adsorption to the membrane may trigger complement activation, coagulation, and inflammation [10,13,14,23,55,56].

##### Investigation of Hemocompatibility in Clinical Studies

Hemocompatibility was also investigated in clinical studies. In total, three studies compared the hemocompatibility profile of the FX CorAL to other dialyzers on the market (Table 2) [39,40,42]. The results for these studies are summarized in Table 5.

All three studies investigated complement activation, platelet activation, as well as immune cell activation during the treatment. eMPORA III also investigated interdialytic changes of hemocompatibility markers [42].

**Figure 4:**
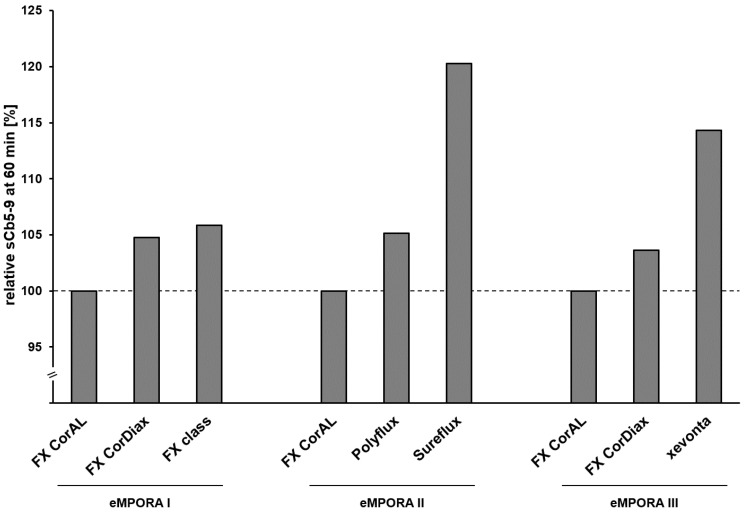
Relative sCb5-9 levels during treatment with of FX CorAL and comparators in the three randomized controlled trials eMPORA I, eMPORA II, and eMPORA III [39,40,42]. FX CorAL is used as reference.

Regarding complement activation, the studies investigated the course of complement factors C5a (eMPORA I and II [39,40]), C3a, and sC5b-9 (all three studies [39,40,42]) during the dialysis sessions at time points 15 min, 60 min, and 240 min as well as before the dialysis treatment. For all dialyzers, the complement factors showed a typical course over time, with a peak value at 15 min for C3a and C5a and at 60 min for sC5b-9. The FX CorAL dialyzer showed the lowest C3a and sC5b-9 peak levels across all three studies. Figure 4 shows the relative sC5b-9 peak levels at 60 min compared to the FX CorAL dialyzer. The increases of the complement factor levels were in most cases significantly lower for the FX CorAL dialyzer as compared to the other investigated dialyzers (Table 5).

Regarding platelet activation, all three studies investigated platelet counts during the dialysis sessions at time points 15 min, 60 min, and 240 min as well as before the dialysis treatment. For all dialyzers and all studies, platelet counts dropped after treatment start, as typically observed during dialysis treatments [69]. While the platelet drop with the FX CorAL was numerically lower in most comparisons with other polysulfone- or polyethersulfone-based dialyzers, statistical significance was only reached vs. Polyflux at 240 min (Table 5). However, when investigating β-Thromboglobulin (β-TG), as a sensitive marker of platelet activation, eMPORAIII showed a clear and significant differentiation between the FX CorAL and the other comparators, with the FX CorAL treatments having the lowest β-TG levels throughout the session. In contrast, Thromboxane B2 (TxB2), which is also a marker of platelet activation showed inconsistent findings, which could be caused by its strong elimination during treatment due to the low molecular weight (371 Da). Regarding plasmatic coagulation activation, eMPORA I and eMPORA II investigated Thrombin-antithrombin III (TAT) but could not find any significant differences between the different dialyzers.

Regarding cell activation, inflammation, and oxidative stress, also different markers were investigated in the three clinical studies during the treatment sessions (Table 5). Leukocytes typically show a drop in their cell counts after treatment start [70], which was also observed in the eMPORA studies, and which is a marker of cell activation during dialysis treatments. The FX CorAL generally induced a lower leukocyte drop 15 min after treatment start, pointing towards lower immune cell activation. In line, FX CorAL also showed lower levels of Polymorphonuclear (PMN) elastase and Leukotriene B4 (LTB4), two markers of leukocyte activation, in eMPORA II and eMPORA III studies. All other investigated markers of cell activation, inflammation and oxidative stress did not differ between the different dialyzers (Table 5).

#### 3.2.4. Investigation of Safety

##### Investigation of Safety in Clinical Studies

An essential requirement for each novel dialyzer is its safe use during dialysis treatments. Therefore, investigation of safety in clinical studies is a critical part and has also been part of all studies performed with the FX CorAL dialyzer.

Adverse events (AEs), which are evaluated in the context of such clinical studies, can be categorized based on their seriousness (serious vs. non-serious), relation to the medical device (i.e., dialyzer) and/or medical procedure (i.e., dialysis treatment), and whether they are expected or unexpected.

Serious adverse events (SAEs) are distinguished from non-serious adverse events (nsAEs) by their life-threatening nature, requirement for hospitalization, or significant medical consequences. AEs directly linked to the dialyzer and/or medical procedure are classified as adverse device effects (ADEs) and may include issues arising solely from the device or medical procedure or both. Expected ADEs are those anticipated based on prior clinical knowledge or device specifications, while unexpected events represent unanticipated reactions.

Table S1 gives an overview of AEs including the respective numbers of AEs in the four clinical studies [39,40,41,42], summarized for all investigated dialyzers.

To determine the safety profile of the FX CorAL dialyzer, particular focus was placed on collecting and analyzing (S)ADEs related to dialyzers alone or in combination with medical procedure reported across all four studies. Figure S1 illustrates known (S)ADEs in dialysis along with their clinical manifestations (medical conditions and symptoms).

ADE types occur with varying frequency and severity and significantly impact patient outcomes [71,72,73,74,75,76,77].

Among the most common ADEs are fluid- and electrolyte imbalances, which result from improper ultrafiltration or ionic clearance during dialysis sessions [78,79,80]. These issues can manifest as hypovolemia or hypervolemia, leading to symptoms such as dizziness, fatigue, headache, edema, or even cardiac arrhythmias in severe cases [79,81,82]. Intradialytic hypotension, occurring in up to 10–12% of dialysis sessions, is typically caused by excessive fluid removal, resulting in reduced blood pressure leading to symptoms like dizziness, fainting, nausea, vomiting, or shock in severe cases [83,84,85]. Electrolyte disturbances, including hypokalemia or hyperkalemia, are also frequent and may cause muscle cramps, weakness, headache, or life-threatening arrhythmias [86,87,88].

Hemocompatibility issues, including clotting within the dialyzer, are another prevalent complication [89,90]. Insufficient anticoagulation or patient-specific factors often contribute to clotting, reducing treatment efficiency and potentially causing anemia, fatigue, or thrombogenicity [91]. Hemolysis, though less common, arises from mechanical stress or improper dialysis settings [92,93]. It presents with symptoms such as dark urine, jaundice, or, in severe cases, circulatory shock [94].

Biocompatibility issues related to the dialyzer materials include allergic reactions and endotoxin-related pyrogenic reactions. Allergic reactions, which are relatively rare, represent a subset of hypersensitivity reactions and are caused by an IgE-mediated immune response to the dialyzer membrane or sterilization agents that begin a few minutes after starting dialysis [95,96]. Symptoms can range from mild itching and rash to severe anaphylaxis [97]. Regarding dialyzer materials, some previous studies speculated that PVP could be a possible cause for the rarely occurring hypersensitivity reactions during treatments with synthetic dialyzers [98,99,100,101]. Typically, these patients are then switched to cellulose-based or polymethylmethacrylate (PMMA) dialyzers for the next treatments. Whether dialyzers, which do not elute PVP into patient’s blood, such as the FX CorAL, may be an adequate alternative for these patients, needs to be evaluated in the future. Regarding endotoxin retention, pyrogenic reactions can result from inadequate water treatment or filter contamination [102,103,104]. These reactions manifest as fever and chills and may exacerbate preexisting inflammation or lead to sepsis in vulnerable patients [77].

Vascular access complications, including infections or thrombosis are another significant concern [105,106,107,108]. Infections at the access site, such as arteriovenous fistulas or central venous catheters, can lead to systemic sepsis, with fever, chills, and tachycardia being common indicators [109]. Thrombosis or stenosis may cause access failure, pain, or swelling, requiring surgical intervention [107,108]. Hematomas and air embolisms, though rarer, can also arise, leading to discomfort or critical symptoms like syncope and dyspnea [75,110].

Device-related mechanical failures, while less common, include blood loss due to circuit disconnections, hemolytic reactions mechanically induced by kinked dialysis lines, pump malfunctions causing insufficient blood flow, and improper temperature of the dialysate or blood circuit, or sensor failures affecting ultrafiltration and blood pressure monitoring [92,111,112]. Such events can compromise treatment and result in feeling cold or in severe symptoms like anemia or intradialytic hypotension.

Dialysis disequilibrium syndrome is one of the rarest but most severe complications, arising from rapid solute removal that leads to osmotic shifts and cerebral edema [75,113,114]. Symptoms include headache, nausea, vomiting, seizures, and in extreme cases, coma.

##### Investigation of Safety in FX CorAL Studies

1. Safety population and methodology

The analysis presented in this article is a summary of safety information reported in the four clinical studies with the FX CorAL dialyzer [39,40,41,42]. The total number of study patients and treatment sessions for this safety analysis across all investigated dialyzers was 249 and 4149, respectively. The number of patients and treatments for the investigational device FX CorAL and the 6 comparator dialyzers are given in Table S2.

In all studies, adverse events were classified according to the MedDRA Preferred Terms (PT) and assigned to broader Level 1 categorization terms based on clinical context, seriousness, severity, relationship to the dialyzer and/or medical procedure, expectedness, and patient outcomes, as described before. The frequencies of adverse events were calculated based on the number of study patients and the number of treatments administered.

2. ADE Types and Frequency

Among the total safety population of 249 study patients and 4149 treatment sessions (Table S2), a total of 446 adverse events were documented, categorized as either serious (SAEs, *n* = 27) or non-serious (nsAEs, *n* = 419) (Table S1). Of these 446 adverse events, 94 were linked to the dialyzer and/or medical procedure (ADE: 93/SADE: 1). A total of 39 study patients (15.7% of the total population) experienced at least one ADE.

Most ADEs were solely related to the medical procedure, and only a lower frequency was related to the dialyzer or to both (Table S1). Of 94 (S)ADEs across the four clinicals studies, 46 AEs were linked solely to the medical procedure, while 14 AEs were related exclusively to the dialyzer, and an additional 34 AEs were related to the medical procedure and the dialyzer. These results are in line with the literature, which identified dialysis procedure per se as the most common cause for ADEs [73,74,75,76,77,85,115]. It is important to note that ADEs in dialysis treatments are often multifactorial, involving both the dialyzer and the procedure (Figure S1).

The respective adverse events according to MedDRA Preferred Terms (PT) are listed in Table 6. The most frequently observed Level 1 adverse event category was electrolyte imbalance and include events such as muscle spasms, myalgia, vomiting, malaise, pain, asthenia, palpitations, hyperphosphatemia, or hyperkalemia. Muscle spasms were the most reported adverse event in this category, observed in 7.2% of patients (*n* = 18) and in 0.4% of treatments. Across all studies, electrolyte imbalance occurred 41 times, leading to a frequency of 16.5% among all study patients and of 1.0% among all study treatments. The second most common observed Level 1 adverse event category was fluid imbalance with 27 occurrences, equating to 10.8% of patients and 0.7% of treatments. Intradialytic hypotension was the most common adverse event in the fluid imbalance category, affecting 3.6% of patients (*n* = 9) and occurring in 0.2% of treatments, a frequency notably lower than the 10–12% reported in the literature [84]. The other findings are consistent with the types and frequency categories of AEs typically associated with dialysis treatments, where large fluid shifts and electrolyte corrections are inherent to the procedure [115]. However, some symptoms, like ear pain, flatulence, and defecation urgency, also might be due to other underlying medical conditions. The other observed Level 1 adverse event categories had a much lower frequency, ranging from 0.4% to 2.4% of study patients and 0.1% or lower of study treatments. The absence of pyrogenic reactions, microbial contaminations and allergic reactions was a positive deviation from literature data [77,102,103,104], reflecting effective sterilization and infection control measures.

Importantly, none of the ADEs with relationship to the dialyzers were classified as serious (Table S1), indicating that no events led to significant deterioration in health or required major medical interventions. One ADE with relationship to the medical procedure was classified as serious, linked to the level 1 term electrolyte imbalance (MedDRA PT: hyperkalemia), and occurred during the treatment sequence with the xevonta dialyzer. In general, the severity of the ADEs was predominantly mild, with a few instances of moderate severity, particularly related to electrolyte and fluid imbalances.

Moreover, in line with the previous literature, all identified ADEs in the present studies were expected (Table S1), meaning they were expected as potential outcomes of dialysis treatments.

The outcomes of all reported ADEs were favorable, with all events either resolving completely or being ongoing without serious or fatal outcomes. No cases resulted in severe long-term effects or sequelae, further supporting the safety profile of the HDF procedures and the dialyzers used in these studies.

3. FX CorAL safety profile

When analyzing the safety profile specifically for the FX CorAL dialyzer among all 1389 treatments, eight ADEs were related to the dialyzer alone (0.6% of study treatments) (Table 6). All these ADEs were attributed to two patients among the total analysis population (*n* = 241). Five more ADEs were related to the dialyzer and medical procedure. The majority of these ADEs were classified as fluid imbalance (*n* = 8), followed by electrolyte imbalance (*n* = 2). No ADE was attributed to hemocompatibility-related complications, such as chronic inflammation or immune responses. The severity of the ADEs was uniformly mild to moderate. Importantly, there were no unexpected new risks or complications associated with the use of the FX CorAL dialyzer. The measures to treat these related AEs were well-established, and all patient outcomes were favorable, with ADEs resolving completely. When compared to the grouped comparator dialyzers (xevonta, FX CorDiax, SUREFLUX, Polyflux, ELISIO, FX class), the FX CorAL showed comparable safety outcomes (frequency per treatment: FX CorAL 2.1%; other dialyzers: 2.4%), with consistently low rates of device-related adverse events and no significant differences in electrolyte or fluid imbalance occurrences. In line, the FX CorAL dialyzer did not differ in patient-reported outcomes (PROs) such as sleep quality, fatigue, or pruritus, either positively or negatively, when compared to the other dialyzers included in the eMPORA III study [42]. Altogether the FX CorAL demonstrated a high level of safety and efficacy, the benefit/risk profile is highly favorable.

## 4. Conclusions and Future Directions

In the present article, we summarized the path which is needed to develop of a new dialyzer including in vitro and clinical evidence generation. Dialyzer innovation helps to improve the life of patients with ESKD, as further development in dialyzer technology—especially membrane technology—may lead to better removal of uremic toxins and to a lower rate of complications and side effects.

We used the example of the new FX CorAL dialyzer with its new Helixone *hydro* membrane, which features enhanced hydrophilicity of the blood-side surface, leading to reduced protein adsorption and subsequently to sustained performance and improved hemocompatibility profile as compared to commonly used dialyzers in clinical practice. The dialyzer was tested in multiple in vitro studies and randomized controlled trials across various patient populations, including those with diverse comorbid conditions such as diabetes and hypertension, validating its suitability for routine treatment of ESKD.

Advances in dialyzer performance and hemocompatibility may contribute to better patient outcomes by potentially reducing the long-term risks associated with dialysis, such as cardiovascular events. Comprehensive safety analyses of the four clinical studies underline the FX CorAL’s robust safety profile, characterized by a low frequency of dialyzer-related adverse events and an absence of unexpected and severe complications reported across thousands of treatment sessions. As FX CorAL undergoes real-world post-market surveillance, continued evaluation of its safety profile confirms its suitability for broader patient groups from different geographical regions. These data will also help to evaluate whether the FX CorAL may be an adequate alternative for patients suffering from hypersensitivity reactions during treatments with synthetic dialyzers and which currently are switched to cellulose-based or PMMA dialyzers. Looking further ahead, longitudinal clinical data will be essential to investigate any long-term benefits in terms of patient survival and quality of life, especially in the context of hemodiafiltration treatments [53,116,117]. Innovation in dialysis care is essential to improve well-being of ESKD patients, bringing hope for more effective, safer, and personalized treatment options worldwide.

## Figures and Tables

**Table 1:** Overview of in vitro studies performed with the FX CorAL dialyzer.

Study Title	Comparators	Investigations	Study Findings for FX CorAL	Study Reference
Complement activation by dialysis membranes and its association with secondary membrane formation and surface charge	FX CorAL FX CorDiax xevonta Polyflux ELISIO FDX THERANOVA SUREFLUX	Hemocompatibility (complement activation) Surface charge Protein adsorption (Albumin sieving coefficient decrease over time)	Lowest C3a, C5a and sC5b-9 activation Most neutral surface charge Lowest albumin sieving coefficient decrease over time (i.e., protein adsorption)	Melchior et al. (2021) [14]
Polyvinylpyrrolidone in hemodialysis membranes: Impact on platelet loss during hemodialysis	FX CorAL FX CorDiax xevonta ELISIO Polyflux THERANOVA	PVP content PVP elution Hemocompatibility (platelet loss)	Highest PVP content Lowest PVP elution (together with FX CorDiax) Lowest platelet loss	Zawada et al. (2021) [13]
Time-resolving characterization of molecular weight retention changes among three synthetic high-flux dialyzers	FX CorAL ELISIO xevonta	Sieving coefficients over time (β2-m, myoglobin, albumin) Molecular weight retention curves over time Effective pore radius over time Predicted albumin loss	Highest β2-m sieving coefficient and lowest decrease in albumin sieving coefficient over time Lowest shift in molecular weight retention curves over time Lowest reduction in effective pore radius over time Low predicted albumin loss	Zawada et al. (2022) [12]
Impact of Hydrophilic Modification of Synthetic Dialysis Membranes on Hemocompatibility and Performance	FX CorAL FX CorDiax	Contact angle (hydrophilicity) PVP elution during shelf-life	Lower contact angle (i.e., higher hydrophilicity) No PVP elution during complete shelf-life (3 years)	Zawada et al. (2022) [10]
Hydrophilic Modification of Dialysis Membranes Sustains Middle Molecule Removal and Filtration Characteristics	FX CorAL FX CorDiax xevonta Diacap Pro HF ELISIO DORA Revaclear Cellentia	Clearance over time (β2-m, inulin, creatinine, urea) TMP/filtrate flow over time	Highest β2-m clearance after protein fouling Lowest TMP increase/filtrate flow decrease over time	Zawada et al. (2024) [11]

C3a: complement factor C3a; C5a: complement factor C5a; sC5b-9: complement factor sC5b-9; β2-m: β2-microglobulin; TMP: transmembrane pressure. Diacap Pro: B.Braun, Melsungen, Germany; HF: Wego, Weihai, China; DORA: Bain Medical, Guangzhou, China; Revaclear: Baxter, Deerfield, US; Cellentia: Nipro, Osaka, Japan.

**Table 2:** Overview of clinical studies performed with the FX CorAL dialyzer.

Study Acronym/ Study Title	Study Design	Therapy	Number of Patients *	Duration	Comparators	Primary Endpoint	Further Endpoints	Study Findings for FX CorAL	Study Reference
**eMPORA I**/ Safety and clinical performance of a dialyzer with a modified polysulfone membrane	Open, multicenter, randomized controlled trial (Germany)	Online-post dilution HDF/ without autosub plus	45	4 weeks	FX CorAL FX CorDiax FX class	β2-m removal rate (non-inferiority of FX CorAL)	Removal rate and clearance of myoglobin and small molecules Hemocompatibility makers Safety	Non-inferior in β2-m removal rate vs. both comparators Lower complement activation vs. FX class Comparable safety profile	Kempkes-Koch et al. (2021) [39]
**eMPORA II**/ Comparative safety and clinical performance of dialyzers applied during post-dilution online hemodiafiltration	Open, multicenter, randomized controlled trial (Germany)	Online-post dilution HDF/ without autosub plus	58	4 weeks	FX CorAL Polyflux SUREFLUX	β2-m removal rate (non-inferiority of FX CorAL)	Removal rate and clearance of myoglobin and small molecules Hemocompatibility makers Safety	Non-inferior in β2-m removal rate vs. both comparators Lower complement and leukocyte activation Comparable safety profile	Ehlerding et al. (2021) [40]
**comPERFORM**/ Comparative clinical performance of dialyzers applied during high volume online hemodiafiltration	Open, multicenter, randomized controlled trial (Germany)	Online-post dilution HDF/ with autosub plus	52	5 weeks	FX CorAL ELISIO xevonta	β2-m removal rate (non-inferiority/superiority of FX CorAL)	Removal rate and clearance of myoglobin and small molecules Albumin loss Safety	Superior in β2-m removal rate vs. both comparators Low albumin loss Comparable safety profile	Ehlerding et al. (2022) [41]
**eMPORA III**/ Comparison of clinical performance and hemocompatibility of dialyzers applied during postdilution online hemodiafiltration	Open, multi-center, randomized controlled trial (Germany, Czech Republic, Hungary)	Online-post dilution HDF/ without autosub plus	76	14 weeks	FX CorAL FX CorDiax xevonta	β2-m removal rate (non-inferiority/superiority of FX CorAL)	Removal rate and clearance of myoglobin and small molecules Hemocompatibility makers PRO Safety	Non-inferior in β2-m removal rate vs. both comparators; superior vs. xevonta Lower complement, leukocyte and platelet activation Comparable PRO scores Comparable safety profile	Ehlerding et al. (2024) [42]

* according to intention to treat population; β2-m: β2-microglobulin; HDF: hemodiafiltration; PRO: patient reported outcomes. FX class: Fresenius Medical Care, Bad Homburg, Germany.

**Table 3:** Patient characteristics and treatment parameters in clinical studies performed with the FX CorAL dialyzer.

	eMPORA I	eMPORA II	comPERFORM	eMPORA III
Patient characteristics *				
Age [years]	66.3 ± 13.6	67.8 ± 13.4	64.8 ± 14.5	67.0 ± 15.6
Gender [% male]	76	74	85	74
Mean BMI [kg/m^2^]	27.7 ± 5.8	28.9 ± 6.6	28.2 ± 7.5	27.3 ± 5.1
Diabetes [%]	31	36	21	34
Median time on RRT [months]	42	50	56	40
Treatment parameter (online post-dilution HDF) **				
Blood flow rate [mL/min]	303 ± 29	306 ± 29	331 ± 12	345 ± 44
Dialysate flow rate [mL/min]	499 ± 5	500 ± 0.1	529 ± 19	577 ± 96
Substitution volume [L]	20.2 ± 5.7	17.6 ± 4.5	25.4 ± 2.8	21.0 ± 3.2
Ultrafiltration volume [L]	1.9 ± 1.1	2.2 ± 1.2	2.0 ± 0.2	2.3 ± 1.0
Effective treatment time [min]	269 ± 29	272 ± 29	273 ± 14	262 ± 21

BMI: body mass index; RRT: renal replacement therapy; * Patient characteristics are presented for the total population in the respective studies; ** Treatment parameters are presented for the treatments with the FX CorAL dialyzer. No significant differences were found between treatments with other dialyzers.

**Table 4:** Primary and secondary performance endpoints in clinical studies performed with the FX CorAL dialyzer.

Performance Parameter	eMPORA I		eMPORA II		comPERFORM		eMPORA III	
	FX CorAL vs.		FX CorAL vs.		FX CorAL vs.		FX CorAL vs.	
	FX CorDiax	FX class	SUREFLUX	Polyflux	xevonta	ELISIO	FX CorDiax	xevonta
β2-m RR [non-inferiority]	★	★	★	★	★	★	★	★
β2-m RR [superiority]	-	-	★	c	★	★	c	★
β2-m clearance	c	★	★	c	★	★	c	★
Myoglobin RR	c	★	#	#	★	★	★	★
Myoglobin clearance	c	★	#	c	★	c	★	★
Phosphate RR	#	#	c	c	c	c	c	c
Phosphate clearance	c	c	c	c	c	c	c	c
Creatinine RR	#	c	c	c	c	c	c	c
Creatinine clearance	c	c	#	c	c	c	c	c
Urea RR	c	c	c	c	c	c	c	c
Urea clearance	c	c	#	#	c	c	c	c
Albumin loss	-	-	-	-	c	c	-	-

β2-m: β2-microglobulin; RR: removal rate; ★: FX CorAL significantly higher vs. comparators; #: FX CorAL significantly lower vs. comparators; c: comparable; -: not determined.

**Table 5:** Overview of hemocompatibility markers in clinical studies performed with the FX CorAL dialyzer.

	Performance Parameter	eMPORA I		eMPORA II		eMPORA III	
		FX CorAL vs.		FX CorAL vs.		FX CorAL vs.	
		FX CorDiax	FX class	SUREFLUX	Polyflux	FX CorDiax	xevonta
Complement activation	C3a	c^15^	c^15^	★^15^	c^15^	★^15^	★^15^
	C5a	c^15^	★^15^	★^15^	c^15^	-	-
	sC5b-9	c^15^	★^15^	★^60^	★^60^	c^60^	★^60^
Cell activation/ inflammation	Leukocyte counts	c^15^	c^15^	★^15^	★^15^	★^15^	c^15^
	Monocyte counts	c^240^	c^240^	c^240^	c^240^	c^15^	★^15^
	Neutrophile counts	c^240^	c^240^	c^240^	c^240^	★^15^	c^15^
	PMN elastase	c^240^	c^240^	c^240^	★^240^	★^60^	★^60^
	LTB4	-	-	-	-	★^15^	c^15^
	IL-6	-	-	-	-	c^AUC^	c^AUC^
	IL-8	-	-	-	-	c^AUC^	c^AUC^
	sICAM-1	-	-	-	-	c^240^	c^240^
	hsCRP	c^240^	c^240^	-	-	c^0^	c^0^
Platelet activation/ coagulation	Platelet counts	c^240^	c^240^	c^240^	★^240^	c^15^	c^15^
	β-TG	-	-	c^240^	c^240^	★^60^	★^60^
	TxB2	-	-	-	-	c^15^	#^15^
	TAT	c^240^	c^240^	c^240^	c^240^	-	-
Oxidative stress	MDA	-	-	-	-	c^AUC^	c^AUC^
	GSH-Px activity	-	-	-	-	c^AUC^	c^AUC^
Contact phase activation	Kallikrein	c^240^	c^240^	c^240^	c^240^	-	-

PMN elastase: Polymorphonuclear elastase; LTB4: Leukotriene B4; IL-6: Interleukin 6; IL-8: Interleukin 8; sICAM-1: Soluble intercellular adhesion molecule-1; hsCRP: high-sensitivity C-reactive protein; β-TG: β-Thromboglobulin; TxB2: Thromboxane B2; TAT: Thrombin-antithrombin; MDA: Malondialdehyde; GSH-Px: glutathione peroxidase; ★: FX Coral significantly better vs. comparator; #: comparator significantly better vs. FX CorAL; c: comparable; -: not determined; superscript numbers indicate the timepoint of measurement reported in the respective study: 0: pre-treatment, 15: 15 min after treatment start, 60: 60 min after treatment start; 240: 240 min after treatment start; AUC: area under the curve over 0–240 min of treatment.

**Table 6:** Frequency of (S)ADEs in clinical studies performed with the FX CorAL dialyzer.

Level 1 Term	Adverse Event (MedDRA PT)	Total Occurrences [Device Related/ Procedure Related/Both]	Frequency Per Patient [%] * [Device Related/ Procedure Related/ Both]	Frequency Per Treatment [%] ** [Device Related/ Procedure Related/ Both]	Occurrences Per Device [Device Related/Procedure Related/Both]	
					FX CorAL ***	Other Dialyzers ****
Electrolyte imbalance	Muscle contractions involuntary Palpitations Vomiting Hyperkalemia Muscle spasms Hyperphosphatemia Blood phosphorous increased Ear pain Abdominal pain Pain in extremity Flatulence Myalgia Hyponatremia Asthenia Malaise Defecation urgency	41 [3/27/11]	16.5 [1.2/10.8/4.4]	1.0 [0.1/0.7/0.3]	11 [2/9/0] Frequency per treatment: 0.8%	30 [1/18/11] Frequency per treatment: 1.1%
Fluid imbalance	Dyspnea Hypovolemia Hypotension Dialysis hypotension Headache Head discomfort Fatigue Circulatory collapse Dizziness Dehydration Blood pressure decreased Visual impairment	27 [8/9/10]	10.8 [3.2/3.6/4.0]	0.7 [0.2/0.2/0.2]	10 [5/2/3] Frequency per treatment: 0.7%	17 [3/7/7] Frequency per treatment: 0.6%
Pruritus, non-allergic	Pruritus	6 [0/0/6]	2.4 [0/0/2.4]	0.1 [0/0/0.1]	1 [0/0/1] Frequency per treatment: 0.1%	5 [0/0/5] Frequency per treatment: 0.2%
Device malfunction (clotting, thrombosis)	Thrombosis in device Dialysis related complication	5 [0/2/3]	2.0 [0/0.8/1.2]	0.1 [0/<0.1/0.1]	3 [0/2/1] Frequency per treatment: 0.2%	2 [0/0/2] Frequency per treatment: 0.1%
Procedural complication (blood-side)	Hemodialysis complication Vessel puncture site hematoma	4 [0/4/0]	1.6 [0/1.6/0]	0.1 [0/0.1/0]	1 [0/1/0] Frequency per treatment: 0.1%	3 [0/3/0] Frequency per treatment: 0.1%
Peripheral vascular disorder	Feeling hot	3 [2/0/1]	1.2 [0.8/0/0.4]	0.1 [<0.1/0/<0.1]	1 [1/0/0] Frequency per treatment: 0.1%	2 [1/0/1] Frequency per treatment: 0.1%
Vascular hypertensive disorder	Hypertension	2 [0/2/0]	0.8 [0/0.8/0]	<0.1 [0/<0.1/0]	2 [0/2/0] Frequency per treatment: 0.1%	0 [0/0/0] Frequency per treatment: 0%
Device mechanical failure	Device leakage	2 [1/0/1]	0.8 [0.4/0/0.4]	<0.1 [<0.1/0/<0.1]	0 [0/0/0] Frequency per treatment: 0%	2 [1/0/1] Frequency per treatment: 0.1%
Anemia	Anemia	1 [0/0/1]	0.4 [0/0/0.4]	<0.1 [0/0/<0.1]	0 [0/0/0] Frequency per treat-ment: 0%	1 [0/0/1] Frequency per treatment: <0.1%
Vasoconstriction, vascular insufficiency	Feeling cold	2 [0/2/0]	0.8 [0/0.8/0]	<0.1 [0/<0.1/0]	0 [0/0/0] Frequency per treat-ment: 0%	2 [0/2/0] Frequency per treat-ment: 0.1%
Infection	Influenza like illness	1 [0/0/1]	0.4 [0/0/0.4]	<0.1 [0/0/<0.1]	0 [0/0/0] Frequency per treat-ment: 0%	1 [0/0/1] Frequency per treat-ment: <0.1%
Total		94 [14/46/34]	37.8 [5.6/18.5/13.7]	2.3 [0.3/1.1/0.8]	29 [8/16/5] Frequency per treat-ment: 2.1%	65 [6/30/29] Frequency per treat-ment: 2.4%

* Frequency per patient calculation: Occurrences/249 (total study population) × 100%; ** Frequency per treatment calculation: Occurrences/4149 (total study treatments) × 100%; *** FX CorAL: 1389 study treatments; **** Other dialyzers: xevonta, FX CorDiax, SUREFLUX, Polyflux, ELISIO, FX class (2760 study treatments).

## Data Availability

The original contributions presented in this study are included in the article/supplementary material. Further inquiries can be directed to the corresponding author.

## Supplementary Materials

The following supporting information can be downloaded at: https://www.mdpi.com/article/10.3390/membranes15050132/s1, Table S1. Categorization of AEs and overview of AE numbers in the four clinical studies with the FX CorAL dialyzer. AEs are summarized across all studies and dialyzers; Table S2. Number of study patients and treatment sessions for safety analysis stratified by study and dialyzer; Figure S1. Dialyzer and medical procedure related (serious) adverse events ((S)ADEs) associated with dialysis treatments. All listed complications can be linked either to the dialyzer or to the medical procedure or both.

## Abbreviations

The following abbreviations are used in this manuscript:

ADE	Adverse device effects
AE	Adverse event
AFM	Atomic force microscopy
AUC	Area under the curve
BMI	Body mass index
β2-m	β2-microglobulin
β-TG	β-Thromboglobulin
C3a	Complement factor C3a
C5a	Complement factor C5a
ESKD	End-stage kidney disease
GSH-Px	Glutathione peroxidase
HDF	Hemodiafiltration
hsCRP	High-sensitivity C-reactive protein
IL-6	Interleukin 6
IL-8	Interleukin 8
LTB4	Leukotriene B4
MDA	Malondialdehyde
MWCO	Molecular weight cut-off
MWRO	Molecular weight retention onset
nsAE	Non-serious adverse event
PMMA	Polymethylmethacrylate
PMN elastase	Polymorphonuclear elastase
PRO	Patient reported outcomes
PVP	Polyvinylpyrrolidone
RR	Removal rate
RRT	Renal replacement therapy
SAE	Serious adverse event
sC5b-9	Complement factor sC5b-9
sICAM-1	Soluble intercellular adhesion molecule 1
TAT	Thrombin-antithrombin
TMP	Transmembrane pressure
TxB2	Thromboxane B2
XPS	X-ray photoelectron spectroscopy
