# Supplementary material for: Development and Investigation of a New Polysulfone Dialyzer with Increased Membrane Hydrophilicity
Source: Membranes (Basel). 2025 Apr 30;15(5):132. doi: 10.3390/membranes15050132 (PMC12113146; doi:10.3390/membranes15050132)
Supplement: Supplementary file 1 [file membranes-15-00132-s001.zip › membranes-3588733-SI.pdf]

Table S1. Categorization of AEs and overview of AE numbers in the four clinical studies with the FX CorAL dialyzer. AEs are summarized across all studies and dialyzers.

<table>
<tr><th colspan="14">Adverse event (AE) [n=446]</th></tr>
<tr><td colspan="7">Non-serious adverse event (nsAE) [n=419]</td><td colspan="7">Serious adverse event (SAE) [n=27]</td></tr>
<tr><td colspan="2">Related to dialyzer (ADE) [n=14]</td><td colspan="2">Related to medical procedure (ADE) [n=45]</td><td colspan="2">Related to dialyzer and medical procedure (ADE) [n=34]</td><td rowspan="2">Not related to dialyzer or medical procedure [n=326]</td><td colspan="2">Related to dialyzer (SADE) [n=0]</td><td colspan="2">Related to medical procedure (SADE) [n=1]</td><td colspan="2">Related to dialyzer and medical procedure (SADE) [n=0]</td><td rowspan="2">Not related to dialyzer or medical procedure [n=26]</td></tr>
<tr><td>Expected (ADE) [n=14]</td><td>Unexpected (UADE) [n=0]</td><td>Expected (ADE) [n=45]</td><td>Unexpected (UADE) [n=0]</td><td>Expected (ADE) [n=34]</td><td>Unexpected (UADE) [n=0]</td><td>Expected (SADE) [n=0]</td><td>Unexpected (USADE) [n=0]</td><td>Expected (SADE) [n=1]</td><td>Unexpected (USADE) [n=0]</td><td>Expected (SADE) [n=0]</td><td>Unexpected (USADE) [n=0]</td></tr>
</table>

Table S2. Number of study patients and treatment sessions for safety analysis stratified by study and dialyzer.

Study	Dialyzer	Number of study patients (safety population)	Number of study treatments*
eMPORA I [39]	FX CorAL	46	138
	FX CorDiax	46	138
	FX Class	45	135
	**Total**	**46**	**411**
eMPORA II [40]	FX CorAL	69	207
	SUREFLUX	69	207
	Polyflux	68	204
	**Total**	**69**	**618**
comPERFORM [41]	FX CorAL	52	156
	ELISIO	52	156
	xevonta	52	156
	**Total**	**52**	**468**
eMPORA III [42]	FX CorAL	74	888
	FX CorDiax	71	852
	xevonta	76	912
	**Total**	**82**	**2652**
**All studies**	FX CorAL	241	1389
	xevonta	128	1068
	FX CorDiax	117	990
	SUREFLUX	69	207
	Polyflux	68	204
	ELISIO	52	156
	FX class	45	135
	**Total**	**249**	**4149**

*Calculation of study treatments per dialyzer: eMPORA I, eMPORA II, comPERFORM: Study patients x 3 (3 treatments in one week); eMPORA III: Study patients x 12 (12 treatments in four weeks)

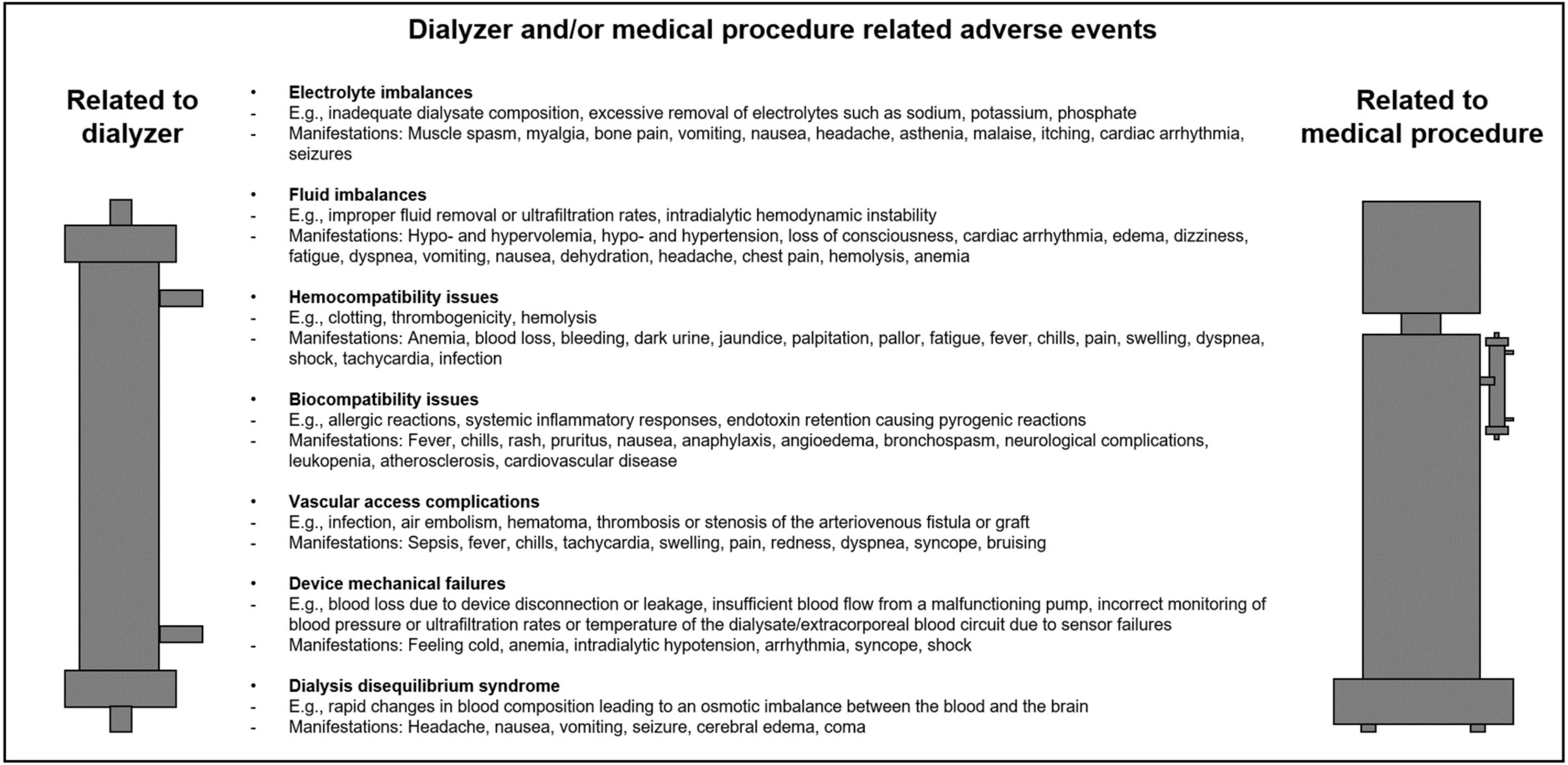

Figure S1. Dialyzer and medical procedure related (serious) adverse events ((S)ADEs) associated with dialysis treatments. All listed complications can be linked either to the dialyzer or to the medical procedure or both.
